# *Taenia crassiceps* Antigens Control Experimental Type 1 Diabetes by Inducing Alternatively Activated Macrophages

**DOI:** 10.1155/2017/8074329

**Published:** 2017-11-08

**Authors:** Arlett Espinoza-Jiménez, Roberto De Haro, Luis I. Terrazas

**Affiliations:** Unidad de Biomedicina, Facultad de Estudios Superiores Iztacala, Universidad Nacional Autónoma de México, Av. De los Barrios 1, Los Reyes Iztacala, Tlalnepantla 54090, MEX, Mexico

## Abstract

Type 1 diabetes (T1D) is an autoimmune disease caused by the selective destruction of the pancreatic *β*-cells, causing inability to produce insulin. Proinflammatory cytokines such as IL-1*β*, IL-6, TNF-*α*, IFN-*γ*, IL-12, IL-17, and NO can be released by CD4 and CD8^+^ lymphocytes as well as by classically activated macrophages (CAM*ϕ*s), which are important in the development of T1D. Helminth infections have been shown to prevent T1D, mainly through Th2-biased responses and increased recruitment of regulatory cell populations. Previously, we have shown that *Taenia crassiceps* infection in mice significantly reduces hyperglycemia, insulitis, and the incidence of T1D. In this study, we determined whether *T. crassiceps-*derived products such as soluble (TcS) or excreted/secreted (TcES) antigens might have a beneficial influence on the development of experimental T1D. Treatment with different doses before or after induction of T1D was analyzed. Mice that were pretreated with TcS were unable to develop T1D, whereas those receiving TcES early after T1D induction displayed significantly reduced insulitis and hyperglycemia along with increased recruitment of alternatively activated macrophages (AAM*ϕ*s) and myeloid-derived suppressor cells (MDSCs). Finally, we examined the modulatory role of AAM*ϕ*s on T1D by depleting macrophages with clodronate-loaded liposomes, demonstrating that AAM*ϕ*s are key cells in T1D regulation.

## 1. Introduction

Type 1 diabetes (T1D) is an autoimmune disease caused by the selective destruction of the pancreatic *β*-cells, lowering insulin production [1]. The absence of insulin disturbs the regulation of blood glucose concentration, resulting in severe hyperglycemia. The onset of diabetes involves both genetic and environmental factors [2]. Proinflammatory cytokines such as IL-1*β*, IL-6, TNF-*α*, IFN-*γ*, IL-12, IL-17, and nitric oxide (NO) can be released by CD4^+^ and CD8^+^ T lymphocytes as well as by CAM*ϕ*s, which seems to be important in the development of insulitis and the death of beta cells [3, 4]. Experimental rodent models such as nonobese diabetic mice (NOD) or multiple low doses of streptozotocin (MLD-STZ) have provided new knowledge and strategies to regulate this disease [5].

During the last few years, anti-inflammatory response has been proposed as a helpful way to regulate the immune system during autoimmune diseases. For example, in NOD mice, the administration of IL-4 or IL-10 may prevent the development of diabetes [6]. In addition, helminth infections have been shown to prevent T1D development. The mechanisms of this effect may consist of inducing a strong Th2-type response and increasing regulatory cell populations, such as Treg, MDSCs, and AAM*ϕ*s, that exert regulatory effects on the immune system of their host [7]. Infections with *Schistosoma mansoni* [8], *Heligmosomoides polygyrus* [9, 10], *Litomosoides sigmodontis* [11], *Trichinella spiralis* [9], *Nippostrongylus brasiliensis*, and *Strongyloides venezuelensis* [12] can provide different levels of protection against the onset of T1D in NOD and MLD-STZ-induced T1D mice, but all these parasites may induce dangerous side effects in their hosts. For example, *Schistosoma mansoni*, *Litomosoides sigmodontis*, and *Trichinella spiralis* have complex life cycles that include migration through several tissues including lungs, bladders, and muscle where these parasites cause damage. Previously, we have shown that infection with the cestode *Taenia crassiceps* significantly decreases hyperglycemia, insulitis and, consequently, the incidence of T1D in mice treated with MLD-STZ. Moreover, higher levels of IL-4 in sera and an increase in the AAM*ϕ* population were induced as well, suggesting that this population could be important in protection against T1D [13].

AAM*ϕ*s are induced by Th2 responses, such as those induced by helminth infections [14]. AAM*ϕ*s can produce IL-10 and TGF-*β* and downregulate levels of proinflammatory cytokines. Additionally, AAM*ϕ*s express suppressive molecules such as PDL-1 and PDL-2 (PD-1 ligands), which are both associated with inhibiting proliferative responses [15–17]. Likewise, AAM*ϕ*s express arginase-1, which induces a shift in arginine metabolism towards the production of L-ornithine, a precursor for polyamines and collagen, important molecules in wound healing [18, 19]. In contrast, CAM*ϕ*s produce iNOS, which converts L-arginine into ROS and NO. Free radicals and NO are critical in inducing damage to *β*-cells [15, 19]. Interestingly, in many studies, infection with helminths or treatment with their antigens led to an increase in the numbers of AAM*ϕ*s. For example, *S. mansoni* infection or treatment with soluble worm antigen (SWA) or soluble egg antigen (SEA) brings about a reduction in the inflammatory response in NOD mice, which has been correlated with increasing AAM*ϕ* and Treg populations [20–22]. In another study, E/S products from *Fasciola hepatica* (FhES) reduced pancreatic islet damage and hyperglycemia in NOD mice, increasing AAM*ϕ*s in the pancreas and pancreatic lymph nodes [23]. Furthermore, adoptive transfer of AAM*ϕ*s induced in vitro by IL-4 and IL-13 in NOD mice decreased hyperglycemia and insulitis, suggesting a protective effect in this population [24]. Similarly, another study using the MLD-STZ model revealed that angiogenesis and the wound healing process were more effective if AAM*ϕ*s were present [25]. However, in most of these studies, helminths or their products were administered before T1D onset, leaving open the question of whether such treatments may be useful as a therapeutic alternative.

Another important population also able to suppress immune responses is the myeloid-derived suppressor cell (MDSC) population. MDSCs are a heterogeneous population of myeloid progenitor cells and immature myeloid cells that delay their maturation and can be differentiated by the expression of different markers such as CD11b and Gr1 [26]. Two subsets of MDSCs were recently defined: monocytic MDSCs, which express CD11b^+^Ly6C^high^Ly6G^−^, and granulocytic MDSCs, which express CD11b^+^Ly6G^+^Ly6C^low^; each population has a different function in cancer, parasitic infections, and autoimmunity [26, 27]. However, their role in T1D development is largely unknown.

The purpose of this study was to determine whether soluble or excreted/secreted products of *T. crassiceps* could protect against T1D induced by MLD-STZ. Additionally, we evaluated the protective role of AAM*ϕ*s and MDSCs induced by *T. crassiceps* and their products in T1D development.

## 2. Materials and Methods

### 2.1. Mice

Six- to eight-week-old male BALB/cAnN mice were purchased from Harlan Laboratories (Mexico) and were maintained in a pathogen-free environment at the animal facility at FES-Iztacala, UNAM, in accordance with institutional and national guidelines.

### 2.2. Parasites and Antigen Preparation/Quantification

Metacestodes of *Taenia crassiceps* were harvested in sterile conditions from the peritoneal cavity of male BALB/cAnN mice after 2–4 months of infection and were washed 4 times with ice-cold, sterile PBS.

- *T. crassiceps* soluble antigen (TcS) was prepared by homogenizing whole metacestodes (10 ml volume) in 2 rounds of 3 seconds each by using a homogenizer (Polytron, Kinematica). The homogenates were centrifuged at 2000 ×g for 20 minutes at 4°C, and the supernatants, which contained PBS-soluble antigens, were collected and frozen at −80°C until further use. Protein concentration was determined using a Bradford protein assay kit (BioRad). Preparation of TcS was similar to that described in [28].
- *T crassiceps* excreted/secreted products (TcES) were prepared as described elsewhere by Terrazas et al. [29]; briefly, metacestodes were harvested in sterile conditions following 3 washes with sterile PBS. Metacestodes were seeded in 6-well plates (Costar, Cambridge, MA) for 24 hours at 37°C and 5% CO_2_. Supernatants were collected and centrifuged at 1000 ×g for 10 minutes. Subsequently, the upper fraction was concentrated using 50 kDa Amicon Ultra Filter tubes (Millipore) and further centrifuged at 2000 ×g for 30 minutes. Protease inhibitors were added to the ≥50 kDa fraction, and samples were stored at −80°C until further use. Protein concentration was determined using a Bradford protein assay kit (BioRad).

### 2.3. Treatments

Different experimental groups as detailed in Table 1 were used.

### 2.4. Blood Glucose Monitoring

Blood glucose was measured at 0, 1, 3, and 6 weeks after T1D induction using an Accu-Check Advantage glucometer (Roche Diagnostics) in animals that had been fasted for 6 hours. Animals were considered diabetic when fasting blood glucose was greater than 200 mg/dl.

#### 2.4.1. Glucose Tolerance Test

At 6 weeks after induction of T1D, mice were subjected to an intraperitoneal glucose tolerance test in order to establish the effects of their diabetes on glucose metabolism. The mice were fasted for 6 hours prior to sample collection. A basal blood sample (time 0) was collected by tail snip, and plasma glucose was evaluated using an Accu-Check Advantage glucometer. Mice were injected i.p. with filtered D-glucose (1.5 mg/kg). Glucose levels were evaluated again at the 30-, 60-, and 120-minute time points.

#### 2.4.2. Histology

Pancreases from all groups were collected 6 weeks after the induction of diabetes. The tissues were processed and embedded in paraffin, and 5 *μ*m sections were cut for analysis. Thin sections were stained with hematoxylin and eosin (H&E) and evaluated microscopically for the presence of insulitis using the following scoring system: noninfiltrated (healthy islets), peri-insulitis (lymphocytes at the periphery of the islets), insulitis 20% (insulitis into the interior of the islets ≤ 20%), and insulitis 40% (insulitis into the interior of the islets ≥ 40% with damage to islet architecture). The insulitis evaluation shown is representative of 10 mice per group (at least 100 islets).

#### 2.4.3. Cytokine ELISAs

Peripheral blood was collected from tail snips at 1, 3, and 6 weeks post induction. Serum IL-4 and TNF-*α* were measured by sandwich ELISA using a commercial kit purchased from Peprotech, Mexico.

#### 2.4.4. Flow Cytometry

Peritoneal exudate cells (PECs) were obtained from the peritoneal cavity from distinct groups of mice 6 weeks p.i. of MLD-STZ. The cells were washed twice with physiological saline solution, and the red blood cells were lysed by resuspending the cells in Boyle's solution (0.17 M Tris and 0.16 M ammonium chloride). Following two washes, the viable cells were counted by trypan blue exclusion with a Neubauer hemocytometer. The PECs were adjusted (1 × 10^6^ cells), and Fc receptors were blocked with anti-mouse CD16/CD32 (Biolegend, CA, USA) and then stained with APC-F4/80, APC-CD11b, FITC-CD11c, FITC-MMR, PE-PDL-1, PE-PDL-2, PE-IL-4R*α*, and PE-Gr1 (all from Biolegend, CA, USA) and incubated for 30 minutes at 4°C in FACSFlow staining buffer (Becton Dickinson). The cells were analyzed using a FACsCalibur and CellQuest Software (Becton Dickinson).

#### 2.4.5. Statistical Analysis

Data were tested for statistical significance using GraphPad Prism software (version 5.0; Graphpad Software, San Diego, CA, USA). For comparisons between the two groups, the Mann–Whitney *U* test was applied to test differences with nonparametric data. Experiments with multiple groups were tested by the Kruskal-Wallis test followed by Dunn's multiple comparison test. Data are presented as mean ± SEM; *p* value < 0.05 was considered statistically significant (^∗^*p* ≤ 0.05, ^∗∗^*p* ≤ 0.01, and ^∗∗∗^*p* ≤ 0.001).

## 3. Results

### 3.1. *T. crassiceps* Soluble Antigen Reduces Experimental T1D in Mice Only If the Treatment Remains Constant

**Figure 1:**
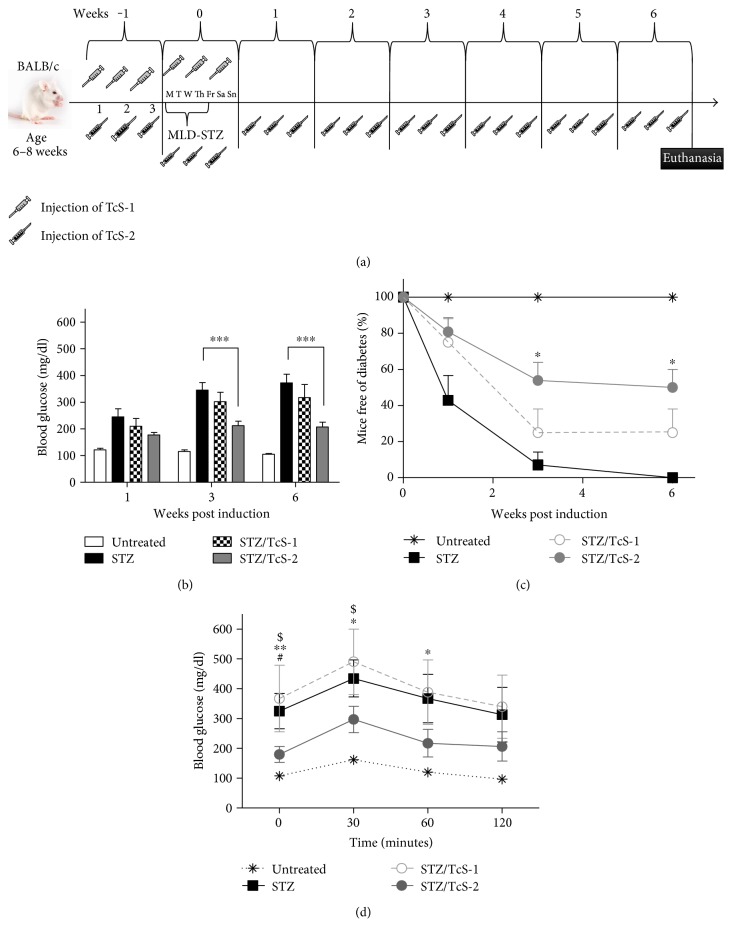
*T. crassiceps* soluble antigens were protective against T1D development only when antigen was constantly injected (STZ/TcS-2). (a) Methodology diagram illustrating treatment with *T. crassiceps* antigen. (b) Blood glucose levels for all groups. STZ/TcS-2 treatment was able to significantly reduce glycemia to normal levels (≤200 mg/dl) compared with STZ group. (c) Percentage of mice free of diabetes; mice with glycemia higher than 200 mg/dl were considered diabetic mice, whereas those with levels below 200 mg/dl were considered free of diabetes. The STZ/TcS-2-treated group showed a lower percentage of T1D incidence than the STZ and STZ/TcS-1 groups. (d) Glucose tolerance test for all groups. ^∗^Differences between STZ versus untreated group. ^#^Differences between STZ/TcS-1 versus untreated group. ^$^Differences between STZ/TcS-2 versus Untreated group. The data represent at least 3 independent experiments; *N* = 5 mice per group. For comparisons between two groups, the Mann–Whitney *U* test was applied to test differences with nonparametric data. Experiments with multiple groups were tested by the Kruskal-Wallis test followed by Dunn's multiple comparison test. Mean ± SEM. ^∗^*p* < 0.05, ^∗∗^*p* < 0.01, and ^∗∗∗^*p* < 0.001.

Our first goal was to test whether soluble *T. crassiceps* antigens may protect against T1D through prior or constant exposure. Thus, mice were treated i.p. with 50 *μ*g of TcS 3 times a week, starting one week before T1D induction and continuing for six consecutive weeks post T1D induction until euthanasia (STZ/TcS-2), or with a dose of 50 *μ*g of soluble antigen one week before induction of MLD-STZ, 3 times in a week (STZ/TcS-1) (Figure 1(a)). Blood glucose was measured once a week.  Figure 1(b) shows that STZ/TcS-2 mice had lower blood glucose levels than STZ at 3 and 6 weeks after disease induction; however, STZ/TcS-1 did not present this effect. Surprisingly, STZ/TcS-2 mice, which had a constant exposure to TcS antigen, downregulated the hyperglycemia at borderline of nonpathological levels (200 mg/dl). Moreover, while the percentage of mice free of diabetes in the STZ group was 0% (with diabetes defined as glucose levels > 200 mg/dl), the STZ/TcS-2 group displayed a significant reduction in T1D (50%), whereas STZ/TcS-1 had a positive effect in only 25% of mice (Figure 1(c)). Additionally, we evaluated glucose tolerance in these mice by injecting them i.p. with D-glucose (1.5 mg/kg), and their blood glucose was evaluated at different times. Hyperglycemia was significantly reduced in mice receiving TcS constantly compared with the STZ and STZ/TcS-1 groups, which showed hyperglycemia up to 300 mg/dl (Figure 1(d)).

### 3.2. Constant Exposure to TcS Reduces Insulitis in T1D-Induced Mice

**Figure 2:**
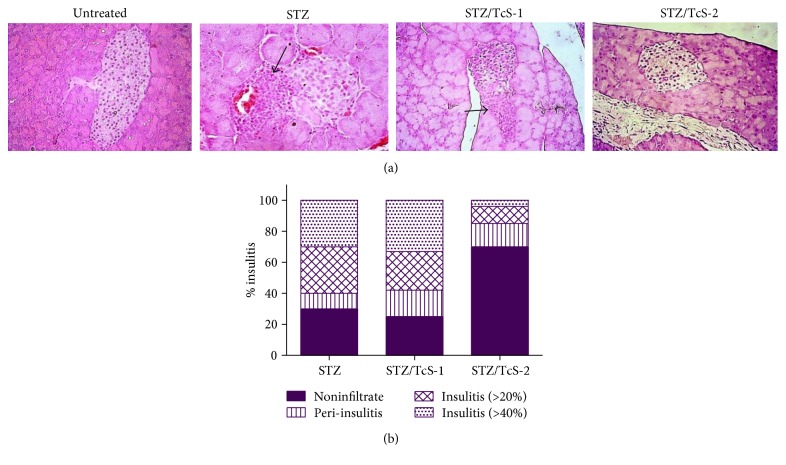
Constant TcS exposure reduces insulitis in MLD-STZ-treated mice. T1D-induced mice were treated with TcS for 6 weeks. The animals were sacrificed, and the pancreases were processed, embedded in paraffin, and cut into 5 *μ*m sections. (a) Representative pictures of islets of Langerhans stained with H&E and evaluated microscopically for the presence of insulitis. Arrows show cellular infiltrate in islets of Langerhans. Magnification of 400x. (b) Percentage of insulitis. Score of infiltrated islets. Noninfiltrated: without damage. Peri-insulitis: infiltration only at the periphery of the islets. Insulitis 20%: infiltration of ≥20% or more but less than 40%. Insulitis 40%: infiltrate of ≥40% of islets. The insulitis evaluation shown is representative of 10 mice per group (at least 100 islets were counted).

At week 6 post T1D induction, mice were euthanized, and their pancreases were removed and processed for histology to evaluate insulitis. The STZ and STZ/TcS-1 groups revealed extensive and severe peri-insulitis and insulitis at the islets of Langerhans, with clear loss of islet architecture, while mice constantly exposed to TcS antigen (STZ/TcS-2) displayed a significant reduction in the number of infiltrated islets and the structure remained unmodified in most of them (Figures 2(a) and 2(b)).

### 3.3. Constant TcS Treatment in Diabetic Mice Induces High Levels of IL-4

**Figure 3:**
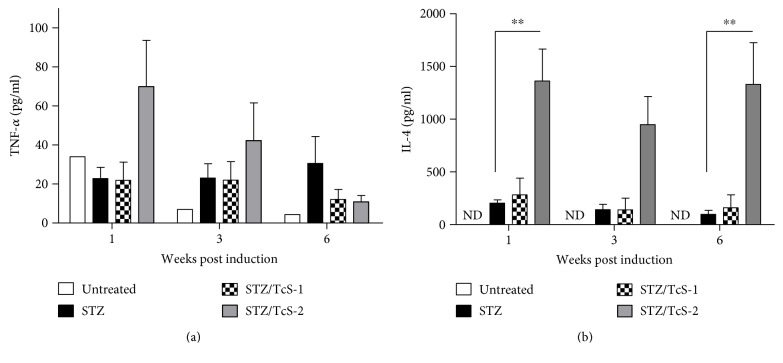
Injection of *T. crassiceps* soluble antigen increases IL-4 in serum. Serum was collected from mice at 1, 3, and 6 weeks p.i. (a) TNF-*α* and (b) IL-4 were detected by ELISA sandwich. ND = nondetected. ^∗∗^*p* < 0.01 by the Mann–Whitney *U* test. Data are representative of two independent experiments. *N* = 4 mice per group.

To evaluate whether TcS treatment might be able to modify inflammatory response, circulating cytokine levels in sera of the different groups were measured. TNF-*α*, an important cytokine related to pancreatic islet damage, displayed similar levels between groups, but at the sixth week after T1D induction, we observed lightly increasing levels of TNF-*α* in STZ mice (Figure 3(a)). In contrast, IL-4 was significantly elevated in STZ/TcS-2 mice since the first week of treatment, whereas STZ and STZ/TcS-1 mice displayed lower levels of this cytokine (Figure 3(b)).

### 3.4. Constant TcS Exposure Recruits AAM*ϕ*s

**Figure 4:**
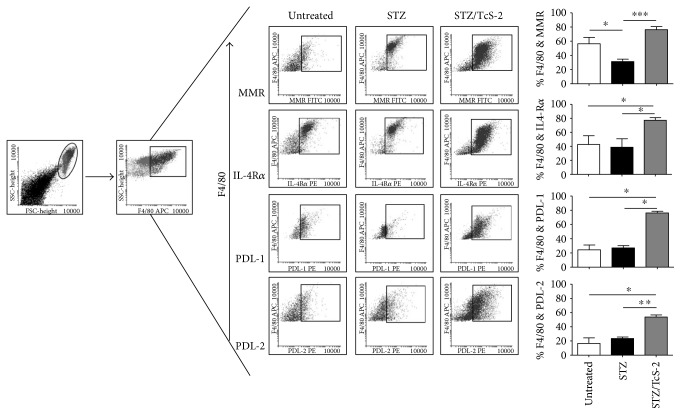
AAM*ϕ*s appear with constant soluble antigen treatment. PECs were obtained at 6 weeks post induction and stained for AAM*ϕ* markers such as MMR, IL-4R*α*, PDL-1, and PDL-2. Its expression was evaluated in F4/80-positive cells by flow cytometry. The data represent at least 2 independent experiments. The data shown are the mean ± SEM. ^∗^*p* < 0.05, ^∗∗^*p* < 0.01 by the Mann–Whitney *U* test, and ^∗∗∗^*p* < 0.001. *N* = 4 mice per group.

Next, we examined whether TcS exposure might favor the recruitment of AAM*ϕ*s. PECs were analyzed by flow cytometry, and we evaluated FSC^high^SSC^high^F4/80^+^ cells to look for AAM*ϕ* markers. As shown in Figure 4, STZ-treated and STZ-untreated mice had reduced percentages of AAM*ϕ*s, while STZ/TcS-2 displayed elevated percentages of AAM*ϕ*s, given that the expression levels of MMR, IL-4R*α*, PDL-1, and PDL-2 were significantly upregulated in STZ/TcS-2-treated mice compared to STZ-treated mice.

### 3.5. Treatment with TcES Reduces the Incidence of T1D

**Figure 5:**
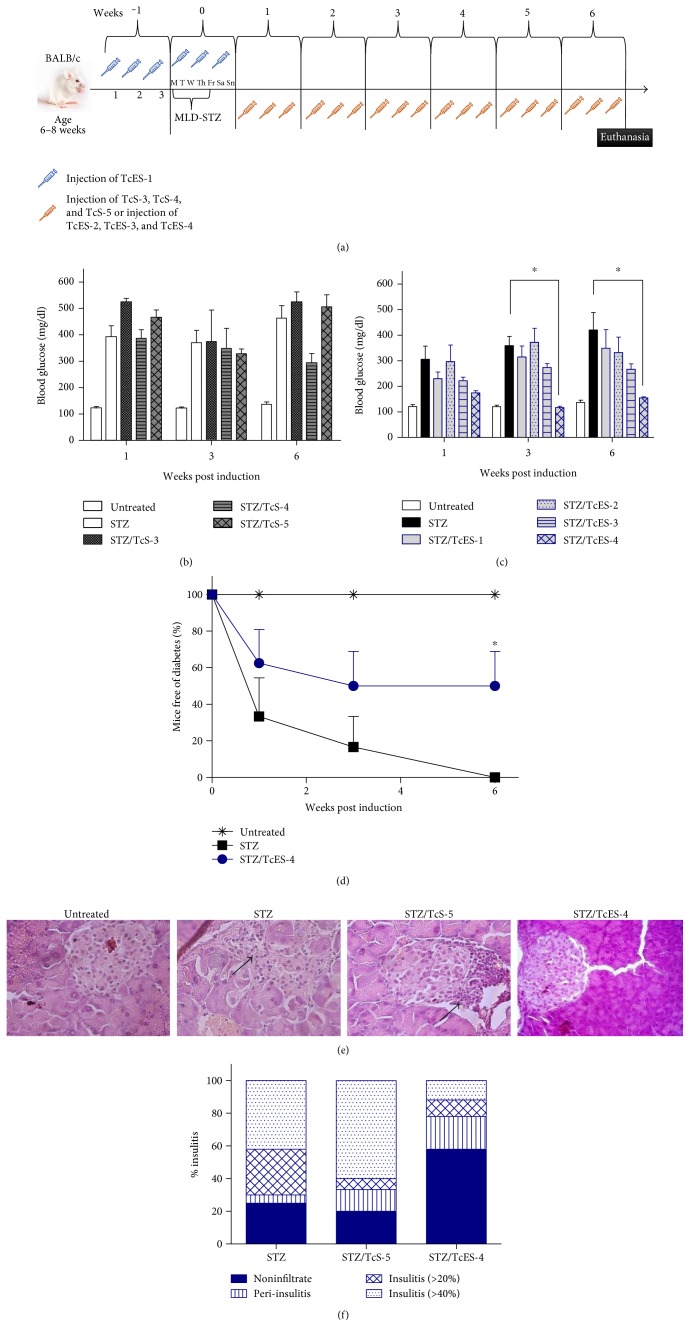
Exposure to TcES reduces T1D damage. Mice were injected with soluble antigen (TcS) or excreted/secreted antigen (TcES). Blood glucose levels were measured for 6 weeks after T1D induction. (a) Methodology diagram illustrating injection of *T. crassiceps* antigen (TcS and TcES). (b) Blood glucose of mice treated with different doses of TcS post T1D induction. (c) Glycemia of mice receiving constant and post T1D induction treatment with TcES. (d) Percentage of mice free of diabetes. Mice with glycemia greater than 200 mg/dl were considered diabetic, whereas those with levels of 200 mg/dl or lower were considered free of diabetes. ^∗^Differences between STZ and STZ/TcES-4. (e) Histology of pancreas. Arrows represent insulitis and pancreatic islet damage. (f) Insulitis score. Noninfiltrated: without damage. Peri-insulitis: infiltration only at the periphery of the islets. Insulitis 20%: infiltration of ≥20% or more but less of 40%. Insulitis 40%: infiltration of ≥40% of islets. Data are representative of 2 independent experiments. *N* = 5 mice per group. For comparisons between two groups, the Mann–Whitney *U* test was applied to test differences with nonparametric data. Experiments with multiple groups were tested by the Kruskal-Wallis test followed by Dunn's multiple comparison test. The data shown are the mean ± SEM. ^∗^*p* < 0.05.

A major question was whether exposure to *T. crassiceps* products early after T1D induction could be effective to protect diabetic mice. We used 3 different variables with the same treatment regime as described before: mice were injected i.p. with 50 *μ*g (STZ/TcS-3), 100 *μ*g (STZ/TcS-4), or 200 *μ*g (STZ/TcS-5) of TcS started in the first week post T1D induction (Figure 5(a)). In addition, we tested TcES treatment in a constant form, 50 *μ*g/dose for 1 week before and 6 weeks after induction of T1D (STZ/TcES-1) or as a post induction treatment 50, 100, or 200 *μ*g beginning after the first week post T1D induction (STZ/TcES-2, STZ/TcES-3, and STZ/TcES-4, resp.) using the scheme described before (Figure 5(a)). We found that TcS treatment did not show a protective effect at any dose when the animals were exposed to *T. crassiceps*-derived antigens one week post induction of diabetes (Figure 5(b)). Also, constant TcES (STZ/TcES-1) treatment was insufficient to reduce hyperglycemia. In contrast, a 200 *μ*g/mouse dose of TcES antigen (STZ/TcES-4) was more effective to reduce the high levels of glucose in diabetic mice in a post induction scheme (Figure 5(c)). Additionally, the percentage of mice free of diabetes in the STZ group at the 6th week was zero, whereas in STZ/TcES-4, the percentage was 50%, as in the STZ/TcS-2 (Figure 5(d)).

Next, we evaluated both insulitis in the pancreas and the damage score in the islets. Figures 5(e) and 5(f) show that the STZ/TcES-4 group had significantly less damage in the pancreas and therefore a lower damage score; most of the islets showed neither infiltration nor peri-insular infiltration, as opposed to the STZ group, which displayed severe damage in the islets and developed insulitis in 20–40%.

**Figure 6:**
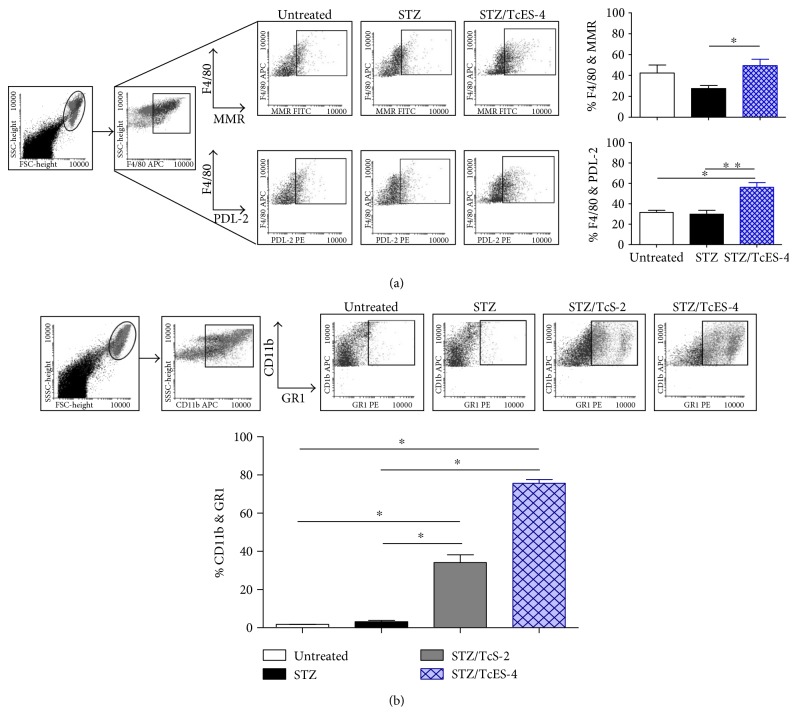
Exposure to TcES during T1D increases both AAM*ϕ* and FSC^high^CD11b^+^ Gr1^+^ populations. (a) PECs were stained for F4/80, MMR, and PDL2 and analyzed in a flow cytometer. (b) PECs were stained for the markers Gr1, CD11c, and CD11b and analyzed by flow cytometry. ^∗^*p* < 0.05 by the Mann–Whitney *U* test. ^∗∗^*p* < 0.01. Data are representative of 2 independent experiments. *N* = 4 mice per group.

STZ/TcES-4 treatment also significantly increased the expression of AAM*ϕ* markers such as PDL-2 and MMR compared to the levels in STZ-induced mice (Figure 6(a)).

### 3.6. Exposure to *T. crassiceps-*Derived Products after T1D Induction Increases the Population of FSC^high^Gr1^+^CD11b^+^ Cells

To examine whether exposure to *T. crassiceps* antigens that were effective in decreasing hyperglycemia (STZ/TcS-2 and STZ/TcES-4) in diabetic mice could modify the recruitment of MDSCs, we evaluated the expression of CD11b and Gr1 markers in peritoneal cells by flow cytometry. As shown in Figure 6(b), STZ/TcS-2 and STZ/TcES-4 treatment led to the recruitment of significantly higher percentages of FSC^high^SSC^high^CD11b^+^Gr1^+^ cells (34% ± 7 and 75% ± 3, resp.), compared with STZ mice (3% ± 0.9) and untreated mice (1.7 ± 0.07%). These results suggest a possible positive role for the CD11b^+^Gr1^+^ population as well as AAM*ϕ*s in regulating T1D development.

### 3.7. Macrophage Depletion Reverses Helminth-Associated Protection against T1D

**Figure 7:**
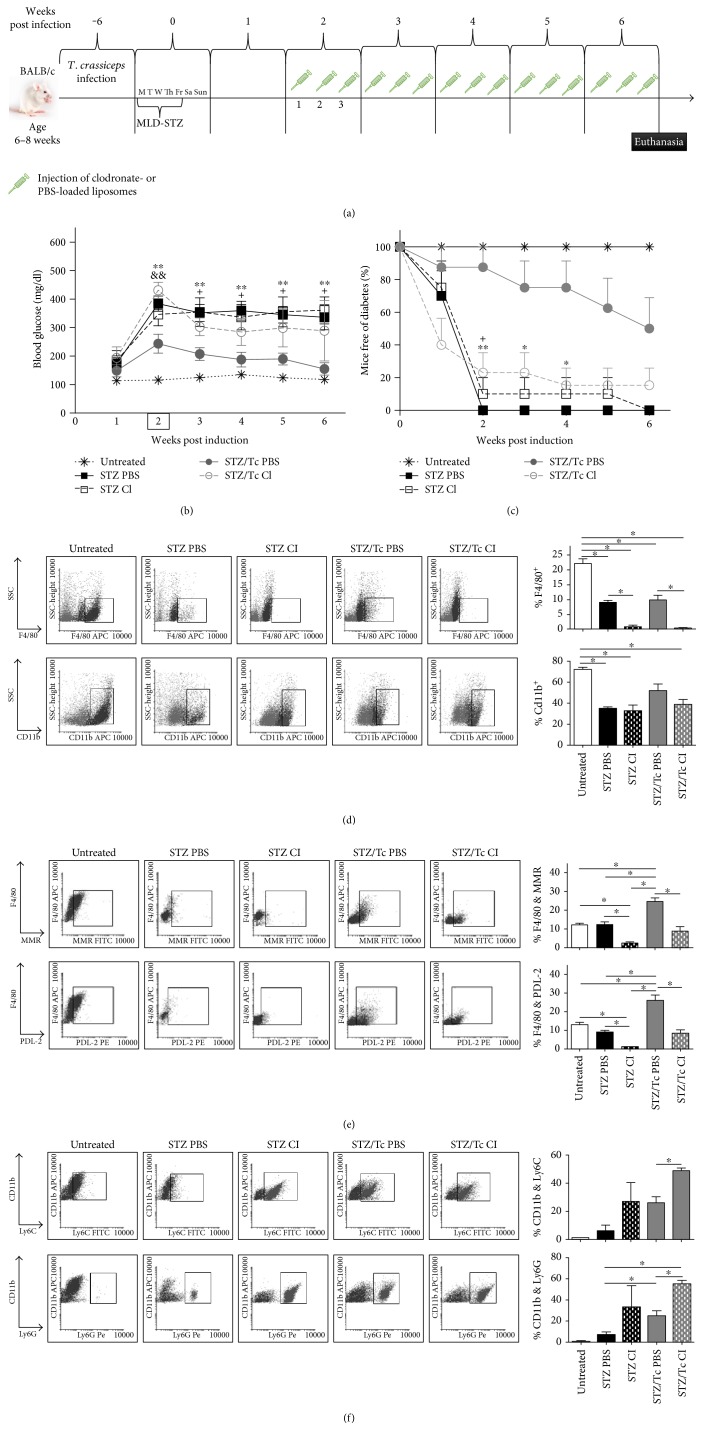
Clodronate treatment reveals a role for AAM*ϕ*s in T1D protection. Mice were infected i.p. by *T. crassiceps* metacestodes. After the sixth week post infection, mice were induced by MLD-STZ and then were injected with clodronate-loaded liposomes or PBS-loaded liposomes, 3 times for a week during four weeks p.i. T1D. (a) Diagram of experimental design. (b) Blood glucose of mice during 6 weeks post T1D induction. (^∗^Differences between STZ/Tc PBS and STZ PBS. ^+^Differences between the STZ/Tc PBS group and STZ Cl. ^&&^Differences between STZ/Tc PBS and STZ/Tc Cl). (c) Mice free of diabetes. Percent of mice free of diabetes (glycemia higher than 200 mg/dl were considered diabetic). ^∗^Differences between STZ and STZ/Tc PBS. (d) Flow cytometry of FSC^high^F4/80^+^ and FSC^high^CD11b^+^ peritoneal cells. (e) Flow cytometry of PECs marked for F4/80, MMR, and PDL-2. (f) CD11b^+^Ly6C^+^ and CD11bLy6G^+^ cells were analyzed in a FACs. For comparisons between two groups, the Mann–Whitney *U* test was applied to test differences with nonparametric data. Experiments with multiple groups were tested by the Kruskal-Wallis test followed by Dunn's multiple comparison test. The data shown are the mean ± SEM. ^∗^*p* < 0.05 and ^∗∗^*p* < 0.01. Data are representative of 2 independent experiments. *N* = 4 mice per group.

To elucidate which cell population was participating in helminth-associated protection against T1D, AAM*ϕ*s or CD11b^+^Gr1^+^, or both, we infected mice with *T. crassiceps* metacestodes, and 6 weeks later, T1D was induced in the infected mice with MLD-STZ. Later, in the second week post T1D induction, diabetic mice were injected i.p. 3 times a week with 2 mg/mouse of clodronate-loaded liposomes or control PBS-loaded liposomes for 6 weeks post T1D induction (Figure 7(a)). Diabetic mice treated with PBS-loaded liposomes (STZ PBS) and clodronate-loaded liposomes (STZ Cl) displayed hyperglycemia beginning in the second week post-T1D induction. In contrast, *T. crassiceps*-infected mice receiving control liposomes (STZ/Tc PBS) displayed levels of blood glucose below 200 mg/dl during whole treatment (Figure 7(b)). Interestingly, when *T. crassiceps*-infected mice received clodronate-loaded liposomes (STZ/Tc Cl), the protective effect of this helminth infection was reversed, and mice turned as diabetic as STZ mice, showing hyperglycemia (Figure 7(b)). The incidence of diabetes was also critically affected by clodronate treatment in each group: all STZ PBS mice displayed hyperglycemia by the second week post T1D induction and were considered diabetic, while the STZ Cl mice showed hyperglycemia until the fourth week post T1D induction. In contrast, in the STZ/Tc PBS group, a protective effect was observed, only 50% of these mice had hyperglycemia (>200 mg/dl), and the rest of the animals remained healthy, but when *T. crassiceps*-infected animals received clodronate liposomes (STZ/Tc Cl), all these animals rapidly turned diabetic, with high levels of glucose at the sixth week, and only 15% were free of diabetes (Figure 7(c)). Finally, to gain further insight about whether AAM*ϕ*s, CD11b^+^Gr1^+^ cells, or both were involved in the protection of diabetic mice and whether clodronate-loaded liposomes depleted any other regulatory cell, flow cytometry of peritoneal cells was performed. We observed that STZ mice receiving PBS-loaded liposomes recruited FSC^high^SSC^high^F4/80^+^ and FSC^high^SSC^high^CD11b^+^ cells in the peritoneal cavity; in contrast, mice receiving clodronate-loaded liposomes displayed a depleted F4/80^+^ cell population but maintained the recruitment of CD11b^+^ cells, demonstrating that clodronate liposomes only depleted macrophages, mostly AAM*ϕ*s, and no other populations such as CD11b^+^ cells (Figure 7(d)).  Figure 7(e) shows that *T. crassiceps* infection in conjunction with STZ significantly increased the markers MMR and PDL-2 compared with all other groups, whereas infected mice that received clodronate liposomes showed a significant reduction in the expression of these AAM*ϕ* markers.

Next, we look for any differences between two different subpopulations of MDSCs, CD11b^+^Ly6C^+^Ly6G^−^ and CD11b^+^Ly6G^+^Ly6C^−^, and whether those populations could change their recruitment when we injected clodronate-loaded liposomes in diabetic animals. Flow cytometry of peritoneal cells shows that all groups expressed CD11b^+^Ly6C^+^ markers at a high percentage except for the untreated group. However, when animals received clodronate liposomes, the percentage of CD11b^+^Ly6C^high^ cells was significantly higher than in mice receiving PBS liposomes (Figure 7(f)). In contrast, granulocytic cells, CD11b^+^Ly6C^−^Ly6G^+^, increased with the injection of clodronate loaded-liposomes; STZ/Tc Cl and STZ/Tc PBS had significant differences compared to mice from the STZ PBS group. Both the STZ/Tc Cl and STZ Cl groups displayed higher percentages of both CD11b^+^Ly6C^+^Ly6G^−^ and CD11b^+^Ly6C^−^Ly6G^+^ cells; all these observations suggest that monocytic and/or granulocytic MDSCs could promote an inflammatory response and damage because both groups showed hyperglycemia and incidence of T1D; and furthermore, the recruitment of AAM*ϕ*s was less evident in these groups. In contrast, STZ/Tc PBS mice maintained AAM*ϕ* recruitment and displayed both reduced glycemia and reduced incidence of T1D, suggesting that AAM*ϕ*s were regulatory cells with an important role in modulating T1D induced by MLD-STZ.

**Figure 8:**
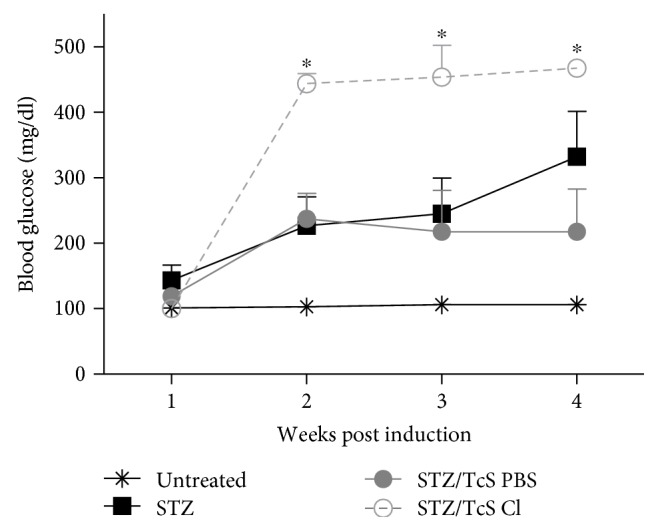
Clodronate treatment abrogates protection against T1D in mice receiving TcS. The clodronate-liposome injections followed the same experimental design as the *T. crassiceps* infection process. Mice were injected with TcS 1 week before T1D induction and continue receiving the treatment for 4 weeks post T1D induction. Blood glucose was measured for 4 consecutive weeks. ^∗^Differences between STZ/TcS Cl and STZ/TcS PBS. Data are representative by 2 independent experiments, each group with 4 mice. Mean ± SEM.^∗^*p* < 0.05 by the Mann–Whitney *U* test.

This last experimental design was replicated with animals exposed to TcS, where mice were injected with TcS one week before T1D induction and for 4 weeks afterward. Data shown in Figure 8 indicate that mice exposed to TcS and treated with PBS liposomes (STZ/TcS PBS) displayed reduced hyperglycemia, whereas mice similarly exposed to TcS but receiving clodronate-loaded liposomes (STZ/TcS Cl) are not protected against increasing blood glucose levels, again supporting the idea that AAM*ϕ*s may play a central role in T1D protection using helminth-derived molecules.

## 4. Discussion

Helminths and their products have been suggested as a powerful weapon against many inflammatory diseases, such as type 1 diabetes, arthritis, colitis, encephalomyelitis, Crohn's disease, and asthma [8, 32–34]; such effects are mainly based on the ability of helminths and their antigens to induce strong Th2-biased responses, with increases in cytokines such as IL-4, IL-10, IL-13, and TGF-*β* [19, 35]. Additionally, they induce regulatory cells, such as AAM*ϕ*s, Treg, and MDSCs, which have been linked with decreased inflammatory responses and less tissue damage as well as with wound healing processes [18, 27, 36, 37].

Type 1 diabetes is an autoimmune disease in which insulin-producing *β*-cells are destroyed by CD4^+^ and CD8^+^ T cells and CAM*ϕ*s [1, 2]. Studies in animal models such as NOD or MLD-STZ-induced T1D mice have provided evidence for the ability of helminths to reduce inflammatory responses, death of *β*-cells, and insulitis [7]. In a previous study, we showed that *T. crassiceps* infection was able to reduce hyperglycemia and insulitis associated with strong recruitment of AAM*ϕ*s; such data suggest that these cells could be important to prevent pancreatic damage and suppress autoreactive T cells [13]. Additionally, our group showed that *T. crassiceps* infection could decrease inflammatory response and damage by autoreactive cells in colitis and experimental autoimmune encephalomyelitis (EAE) [38, 39]. Our goal in the present work was to investigate whether *T. crassiceps* products (TcES or TcS) would mimic the effect of experimental infection by decreasing inflammatory responses induced by T1D, and we looked for cells involved in this regulatory response. Here, we demonstrate that both treatments, TcS and TcES, can regulate hyperglycemia, insulitis, and the incidence of T1D, but with slight differences. We found that it is important for TcS treatment to begin before MLD-STZ induction of T1D and continue with constant injections of TcS for a protective effect. Otherwise, TcES treatment in diabetic mice was more effective to decrease hyperglycemia at a high dose (200 *μ*g), compared with TcS (50 *μ*g), in a post induction system of MLD-STZ-induced T1D. Differences between the effects of TcS and TcES may be explained by a previous work in which exposure of macrophages to TcES modified their inflammatory response to IFN-*γ* through the expression of high levels of SHP-1 and SOCS3, which are suppressors of IFN-*γ*-transducing signaling, but when macrophages were exposed to TcS, these cells did not block IFN-*γ* signaling [40]. In another research, it was found that *T. crassiceps* E/S antigen induced a tolerogenic phenotype in dendritic cells, stopped their maturation, and downmodulated the expression of costimulatory molecules [29]. Such differences observed in vivo and in vitro between TcS and TcES may be associated with differences in the composition or concentration of biomolecules found in the >50 kDa fraction.

Different studies have demonstrated the ability of several helminth-derived products to reduce T1D development; however, most of them were evaluated as a pretreatment for T1D. For example, Zaccone et al. showed that *S. mansoni* products may prevent the development of diabetes in NOD mice, but only if the treatment was started before the fourth week of age [21]. In other research, Amdare et al. showed that treatment of *B. malayi* ES and soluble (adult and microfilaria) product may protect against T1D, but only if the series of injections were started before T1D induction [41]. More recently, in 2016, Ajendra et al. demonstrated that *L. sigmodontis* antigen (crude worm extract) may protect against T1D in later treatments (after 10 weeks of age) in NOD mice, but only as part of a combined therapy with an intranasal proinsulin dosage [42]. In this context, we believe that TcES could be a promising treatment, considering that its effects do not require additional therapy for protection against T1D. Furthermore, with the reasoning that the diagnosis of T1D in humans is reached late, when most of the *β*-cells are destroyed, TcES may prevent or delay pancreatic islet destruction after T1D initiation. In line with this idea, another important question is knowing if *T. crassiceps* antigens can be recognized by human cells; in this regard, Terrazas et.al. showed that human dendritic cells exposed to *T. crassiceps* excreted/secreted antigens displayed a tolerogenic profile [29]. Further studies are needed to prove that TcES may regulate human autoreactive cells as a treatment in T1D in the future.

Another important point about the effect of helminths and their products is their capability to recruit regulatory cells involved in the downregulation of inflammation, as has been demonstrated by several helminth infections (*S. mansoni*, *H. polygyrus*, *B. malayi*, *N. brasiliensis*, *T. spiralis*, *F. hepatica*, and *T. crassiceps)* reducing T1D development; however, all of them were also evaluated as a pretreatment for T1D, and most of them recruited AAM*ϕ*s [8, 9, 11, 12]. Some research has also been focused on products of helminths to search for regulatory cells involved in downregulating inflammation. For example, *S. mansoni* products (SEA and SWA) could induce high numbers of AAM*ϕ*s and Treg cells [20, 21]. E/S products of *F. hepatica* also increased AAM*ϕ* and Treg populations in diabetic mice, suggesting that both populations are related to protection against diabetes [23]. In our previous research, we found that *T. crassiceps* infection in MLD-STZ-induced T1D mice recruited AAM*ϕ*s [13]. Here, we gain knowledge on the role of AAM*ϕ*s during *T. crassiceps* antigen treatment (TcS and TcES) in T1D, where we find increased expression of AAM*ϕ* markers such as MMR, PDL-1, and PDL-2 in T1D-protected mice. These data agree with the recent observation that reduction of hyperglycemia in mice infected with *S. mansoni* was Treg independent, while recruitment of AAM*ϕ*s was important to the process [43]. In that work, the authors blocked Treg cells by injecting a specific anti-Treg antibody, and hyperglycemia did not improve or even turned worse. However, these same authors did not test a specific role for AAM*ϕ*s, whereas in this investigation we show for the first time that AAM*ϕ*s are critical for the protective effect of helminth-derived products in T1D development. Moreover, whereas in the *S. mansoni* infection there was only a limited reduction of hyperglycemia and all the animals became diabetic after 3 weeks, here we showed that *T. crassiceps*-derived molecules or even the whole infection was able to reduce the incidence of T1D by 50% for at least 6 weeks. Together, these data indicate that *T. crassiceps* and their derived molecules display more potent anti-inflammatory activity than that observed in other helminthic infections and that such protective effect is highly dependent on the presence of AAM*ϕ*s.

Additionally, we observed that TcS and TcES exposure directly upregulated the recruitment of CD11b^+^Gr1^+^ cells in diabetic mice compared with STZ and untreated mice. MDSCs have garnered increased interest because they can suppress T cell responses. In 2010, Yin et al. [44] demonstrated that adoptive transference of MDSCs in NOD mice significantly decrease diabetes onset as well as pancreatic islet damage, and these authors suggested that MDSCs might mediate anergy of autoreactive T cells and favor the presence of Treg cells. Conversely, in other research, MDSCs were associated with damage, because these cells were favored differentiation of CD4^+^ cells towards a Th17 profile in the pathogenesis of EAE in mice [45].

To elucidate which cells may have a more significant role downregulating T1D in our system, we decide to deplete phagocytic cells by i.p. injections of clodronate-loaded liposomes during both *T. crassiceps* infection and TcS exposure. Previously, it has been demonstrated that clodronate-loaded liposomes only depleted phagocytic cells (mostly macrophages) in our system of peritoneal injections, without affecting dendritic cells and eosinophils [31]. Here, we found that AAM*ϕ*s were significantly reduced in STZ/Tc Cl mice, while STZ/Tc PBS recruited higher percentages of AAM*ϕ*s. However, we also found that CD11b^+^Ly6C^+^Ly6G^−^ and CD11b^+^Ly6C^−^Ly6G^+^ populations were increased in mice receiving clodronate liposomes (STZ Cl and STZ/Tc Cl), with significant differences from the other groups. Recently, our group demonstrated that high recruitment of CD11b^+^Ly6C^high^ in colitic mice was associated with damage and colon inflammation [38]. In line with this previous report, the present study found a similar outcome in T1D mice, with an elevated number of MDSCs (monocytic and granulocytic) in the peritoneal cavity of mice receiving clodronate, but without protection against T1D development; thus, our data strongly suggest that granulocytic and monocytic cells do not participate as a protective cell population in our system. Further studies are needed to elucidate the role of MDSCs in diabetic mice, such as to demonstrate that these cells have suppressive ability, a key feature to name these cells. Instead, a strong role for AAM*ϕ*s in the anti-T1D effects of *Taenia* products can be assumed; given that STZ/Tc Cl reversed the protective effect of *T. crassiceps* infection, producing similar hyperglycemia levels to STZ and STZ Cl mice and T1D incidence did reach up to 100%, while the STZ/Tc PBS group was protected up to 50%, displaying hyperglycemia levels under 200 mg/dl. A reversal of the protective effect of TcS was also observed when mice exposed to TcS received clodronate liposomes. However, clodronate may also affect CAM*ϕ*s and thus eliminate a dangerous cell population for T1D development, but given the worsening of the glucose levels, this possible fact may support a more critical role for CD8^+^ and CD4^+^ autoimmune cells in tissue damage. Together, these results strongly support the hypothesis that AAM*ϕ*s recruited by *T. crassiceps* infection and *T. crassiceps*-derived products are a key population that importantly reduces inflammatory responses associated with T1D development.

## 5. Conclusion

In conclusion, our findings indicate that exposure to TcS and TcES has a potential protective effect against MLD-STZ-induced T1D development by reducing hyperglycemia and the incidence of this autoimmune disease. Mechanistically, our new data support the hypothesis that AAM*ϕ*s recruited by *T. crassiceps*-derived products play a critical role in downregulating T1D, because when this population is depleted early, the protective effect is abrogated. It is necessary to elucidate the putative signaling pathways triggered by these *T. crassiceps*-derived products to understand more completely the mechanisms associated with their anti-inflammatory and antidiabetic effects.

## Figures and Tables

**Table 1:** Description of experimental groups.

Treatment	Description	Groups
STZ	Diabetic mice were induced with multiple low doses of streptozotocin (MLD-STZ). They received during five days consecutive intraperitoneal injections (i.p) of STZ (Sigma-Aldrich; 45 mg/kg) dissolved in 0.1 M sodium citrate, pH 4.5 [30]	STZ

STZ/TcS	Mice with MLD-STZ and injections of *T. crassiceps* soluble antigens	
	With 50 *μ*g of TcS i.p. 3 times for a week, one week before, and during the week of induction with MLD-STZ	STZ/TcS-1
	Treated constantly with 50 *μ*g i.p. 3 times per week, one week before, during treatment with MLD-STZ, and for 6 weeks post induction until euthanasia	STZ/TcS-2
	Treated with 50 *μ*g i.p. 3 times per week, starting 1 week post induction of T1D and continuing until sacrifice	STZ/TcS-3
	Treated with 100 *μ*g i.p. 3 times per week during 1 week post induction of T1D and for 6 weeks afterward	STZ/TcS-4
	Treated with 200 *μ*g i.p. 3 times per week, starting 1 week post induction T1D and continuing for 6 weeks	STZ/TcS-5

STZ/TcES	Animals treated with injections of *T. crassiceps* excreted/secreted antigens with MLD-STZ	
	Treated with 50 *μ*g i.p. 3 times per week, one week before, during treatment with MLD-STZ, and for 6 weeks post induction	STZ/TcES-1
	Treated with 50 *μ*g i.p. 3 times per week started 1 week post induction of T1D for the rest of the treatment period	STZ/TcES-2
	Treated with 100 *μ*g i.p. 3 times per week since first week post induction of T1D for the rest of the treatment period	STZ/TcES-3
	Treated with 200 *μ*g i.p. 3 times per week, started one week after induction of T1D and during 6 weeks	STZ/TcES-4

Liposomes	Six- to eight-week-old mice were infected i.p. with 20 cysticerci, and then we waited 6 weeks post infection to induce diabetes by MLD-STZ. We chose this time period to induce diabetes because we know by a previous report from our group that the change in the immune response to a Th2 response and the appearance of AAM*ϕ*s have been established to occur in the sixth to eighth week post infection [31]. Macrophages were depleted in vivo using dichloromethylene diphosphonate (clodronate) encapsulated in liposomes. Treatment of liposome injections was followed as Reyes et al. [31]	
	In the second week after T1D-induction, mice were treated with i.p. injections of clodronate liposomes (Cl) or PBS liposomes (200 *μ*l/mouse i.p. 3 times/week) for 5 weeks post T1D induction	STZ Cl STZ PBS
	Two weeks after T1D induction by MLD-STZ, *T. crassiceps*-infected and *T. crassiceps*-uninfected mice were injected i.p. with Cl liposomes and PBS liposomes (200 *μ*l/mouse i.p. 3 times/week) for 5 weeks post T1D induction	STZ/Tc Cl STZ/Tc PBS
	An equivalent treatment with clodronate liposomes was done in TcSA-treated Balb/c mice. Animals were injected on a similar schedule to STZ/TcS-2, the second week before T1D induction and continuing for 4 weeks post induction. Blood glucose levels were measured for 4 weeks	STZ/TcS Cl STZ/TcS PBS

Untreated	Receiving neither antigens nor MLD-STZ	Untreated
